# Transciptome analysis reveals flavonoid biosynthesis regulation and simple sequence repeats in yam (*Dioscorea alata* L.) tubers

**DOI:** 10.1186/s12864-015-1547-8

**Published:** 2015-04-30

**Authors:** Zhi-Gang Wu, Wu Jiang, Nitin Mantri, Xiao-Qing Bao, Song-Lin Chen, Zheng-Ming Tao

**Affiliations:** Zhejiang Institute of Subtropical Crops, Zhejiang Academy of Agricultural Sciences, Wenzhou, 325005 P.R. China; School of pharmacy, Wenzhou Medical University, Wenzhou, 325035 P.R. China; School of Applied Sciences, Health Innovations Research Institute, RMIT University, Melbourne, VIC Australia

**Keywords:** Transcriptome, Flavonoid biosynthesis, *Dioscorea alata* L, Tuber color, Differentially expressed genes, Microsatellite markers

## Abstract

**Background:**

Yam (*Dioscorea alata* L.) is an important tuber crop and purple pigmented elite cultivar has recently become popular because of associated health benefits. Identifying candidate genes responsible for flavonoid biosynthesis pathway (FBP) will facilitate understanding the molecular mechanism of controlling pigment formation in yam tubers. Here, we used Illumina sequencing to characterize the transcriptome of tubers from elite purple-flesh cultivar (DP) and conventional white-flesh cultivar (DW) of yam. In this process, we also designed high quality molecular markers to assist molecular breeding for tuber trait improvement.

**Results:**

A total of 125,123 unigenes were identified from the DP and DW cDNA libraries, of which about 49.5% (60,020 unigenes) were annotated by BLASTX analysis using the publicly available protein database. These unigenes were further annotated functionally and subject to biochemical pathway analysis. 511 genes were identified to be more than 2-fold (FDR < 0.05) differentially expressed between the two yam cultivars, of which 288 genes were up-regulated and 223 genes were down-regulated in the DP tubers. Transcriptome analysis detected 61 unigenes encoding multiple well-known enzymes in the FBP. Furthermore, the unigenes encoding chalcone isomerase (*CHS*), flavanone 3-hydroxylase (*F3H*), flavonoid 3′-monooxygenase (*F3’H*), dihydroflavonol 4-reductase (*DFR*), leucoanthocyanidin dioxygenase (*LDOX*), and flavonol 3-O-glucosyltransferase (*UF3GT*) were found to be significantly up-regulated in the DP, implying that these genes were potentially associated with tuber color formation in this elite cultivar. The expression of these genes was further confirmed by qRT-PCR. Finally, 11,793 SSRs were successfully identified with these unigenes and 6,082 SSR markers were developed using Primer 3.

**Conclusions:**

This study provides the first comprehensive transcriptomic dataset for yam tubers, which will significantly contribute to genomic research of this and other related species. Some key genes associated with purple-flesh trait were successfully identified, thus providing valuable information about molecular process of regulating pigment accumulation in elite yam tubers. In the future, this information might be directly used to genetically manipulate the conventional white-fleshed tuber cultivars to enable them to produce purple flesh. In addition, our SSR marker sets will facilitate identification of QTLs for various tuber traits in yam breeding programs.

**Electronic supplementary material:**

The online version of this article (doi:10.1186/s12864-015-1547-8) contains supplementary material, which is available to authorized users.

## Background

Yam (*Dioscorea alata* L.) is an important tuber crop valued for its dietary carbohydrate, amino acids and essential minerals. It is widely cultivated in tropical and subtropical regions. Most *D. alata* tubers have white flesh, but occasionally, purple-flesh tubers with high anthocyanidin content are produced because of spontaneous variation [1]. Anthocyanins are responsible for the deep purple to red pigmentation of various flowers, fruits, leaves, and other plant tissues [2]. Anthocyanins are perhaps the best characterized flavonoids with studies indicating their important role in plant physiology, in particular, plant defense against herbivores and pathogens. They have also been shown to have multiple health benefits for humans including immunomodulatory, anticancer, cardio-protective, vasodilation, antithrombotic, and UV-protection due to their antioxidant, and anti-inflammatory properties [3]. Therefore, it is no surprise that the purple-flesh yam tubers have recently been selling at a premium price owing to consumer awareness about its health benefits. The current study is aimed at understanding how spontaneous variation leads to anthocyanin synthesis in certain yam strains. Understanding the molecular mechanism of triggering anthocyanin biosynthesis and accumulation in these strains makes it possible to transfer the purple pigment trait to conventional white-flesh cultivar, thus improving the tuber quality and market value.

In the past decade, the flavonoid biosynthesis pathway (FBP) has been well characterized genetically and biochemically in model and non-model plants [4,5]; and a number of genes encoding important enzymes and transcription factors responsible for the FBP have been cloned from a dozen organisms such as *Arabidopsis* [6], grapevine [7], *Petunia* [8], and *maize* [9]. Nevertheless, the complicated mechanism that controls anthocyanin catabolism in different plant species and tissues is far from conclusive. It is reasonable to expect that the loss or accumulation-of-color adaptations in yams are relatively unconstrained because they can be obtained in various ways [10], and affected by multiple intracellular factors such as co-pigmentation [11], pH [12] and metal-chelation in vacuoles [13]. The promotion or suppression of any one of the enzymes catalyzing a series of reactions that make up a pathway will change its final product. For example, Chen et al. [14] found that for independent events causing accumulation of red pigments in variegated peach flowers, a particular subset of genes (*C4H*, *CHS*, *CHI* and *F3H*) were enhanced and co-regulated in the FBP. Lou et al. [15] also revealed that the loss of delphinidin (blue pigment) resulted from the gene suppression of *FLS* and *DFR* in grape hyacinth.

Recently, transcriptomic analysis based on next generation sequencing (NGS) technology has emerged as an extremely powerful method for identifying novel genes associated with biosynthesis of various secondary metabolites in non-model plant species [16,17]. Specially, it has been widely applied to investigate molecular mechanisms of color variation in plant species such as blueberry [18], grape [15], *Brassica Juncea* [19], and potato [20]. In yam, transcription profiles of leaf tissues from one anthracnose susceptible (TDa 95–0310) and two resistant yam genotypes (TDa 87–01091, TDa 95–0328) were analyzed upon infection with the anthracnose fungus; a set of genes involved in defense against anthracnose were identified [21]. Anthocyanins are considered as an important quality trait in yam [22]. However, to date, no effort has been made to uncover molecular basis of different color formation in yam tubers by using RNA-Seq. A previous study by Zhou et al. [23] through RACE technology and RT-PCR analysis, reported that *DaANS1* (a member of *ANS* genes in FBP) controls anthocyanin accumulation of purple-flesh tubers based on its regulation at transcription level. However, a limitation in this study was the use of single cultivar (purple-flesh tuber) and study of one gene (*ANS*). Without comparing the global transcriptional differences between the purple-flesh cultivar and conventional white-flesh cultivar, it is impossible to separate candidate genes related to color formation. Therefore, the molecular mechanism underlying the purple-flesh formation has not yet been fully understood in yam.

Further, being a non-model species, there is lack of genomic resources, in particular, information on SSR markers for marker-assisted breeding (MAS) of yam. Previous genetic inheritance study revealed that some important traits (such as resistance) are controlled by a single dominant locus in yam [24]. Genic-SSR markers appear to be tightly linked to specific gene functions and perhaps even play a direct role in controlling important traits [25,26]. Recent studies have demonstrated the variability in cultivated yam accessions in terms of tuber shape, color, taste and yield [27,28]. However, very limited knowledge is available on the genetic regions associated with these variations, in particular SSR or SNP markers for important genes [29,30]. Therefore, identification of SSR markers from yam transcriptome is crucial for the future of marker-assisted breeding programs.

Here, we report use of RNA-Seq to investigate the transcriptomic differences between yam tubers of a purple-flesh cultivar (DP) and conventional white-flesh cultivar (DW). Differentially expressed genes and their expression patterns were analyzed, and some potential candidate genes responsible for the FBP were successfully identified. We expect this genome-wide transcriptome comparison to provide a novel resource to understand the molecular mechanisms underlying the purple-flesh trait. Moreover, transcriptomic datasets were further exploited to identify a large number of gene-based SSR markers that enable linkage mapping and marker assisted breeding of yams.

## Results and discussion

### Sequencing statistics and assembly

**Figure 1:**
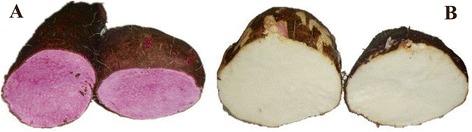
Different pigment expression in yam tubers. **A**: Elite purple-flesh cultivar; **B**: Conventional white-flesh cultivar.

The variation in pigment expression of the purple-flesh cultivar and the conventional white-flesh cultivar of yam is shown in Figure 1. To characterize the transcriptome differences between the two cultivars, two cDNA libraries were prepared from their tubers and subjected to RNA-Seq analysis based on the Illumina HisSeq 2000 platform. After removing adaptors and reads of unknown or low-quality nucleotides, in total, 35,645,052 and 34,585,554 clean reads were respectively obtained from the DP and DW libraries. The information of all high-quality reads has been deposited in the Sequence Read Achieve (SRA) database under the accession ID SRX652481 for DP, and SRX652483 for DW. The high-quality reads from the two libraries were subsequently *de novo* assembled into 125,123 unigenes using Trinity program; the size distribution of these unigenes is shown in Additional file 1. As a result, the *in silico* assembled unigenes ranged from 200 to 14,799 bp with an average length of 592 bp; the N50 value was 875 bp and total size was approximately 71.8 Mb. Furthermore, in order to estimate the efficiency of short-read usage during the *de novo* assembling, we mapped our RNA-Seq reads to the assembled unigenes using TopHat analysis package. A total of 29,676,058 and 29,022,640 sequences from DP and DW library respectively were matched (~80%) (Table 1), indicating that the set of assembled unigenes is applicable to carry out the downstream analysis.

**Table 1:** **Number of mapped reads to the assembled unigenes of yam**

**Item**	**DP**	**DW**	**Total**
Raw bases (bp)	5,624,450,100	5,506,892,100	11,131,342,200
Clean bases (bp)	5,270,942,114	5,105,288,057	10,376,230,171
Raw reads (N)	37,496,334	36,712,614	74,208,948
Clean reads (N)	35,645,052	34,585,554	70,230,606
Total alignment (percent of clean reads) (N, %)	29,676,058 (83.25%)	29,022,640 (83.92%)	58,698,698 (83.58%)
Unique matches (percent of clean reads) (N, %)	23,687,732 (66.45%)	23,203,663 (67.09%)	46,891,395 (66.77%)
Multi-position match (percent of clean reads) (N, %)	5,988,326 (16.80%)	5,818,977 (16.83%)	11,807,303 (16.81%)

Currently, the yam EST library found in Genbank database (http://www.ncbi.nlm.nih.gov/nucest/?term=*Dioscorea*+*alata*) contains 44,134 ESTs from leaves of three genotypes differing in resistance to anthracnose disease [21]. To estimate the level of transcript coverage in this study, we downloaded these ESTs from GenBank and compared them to our transcriptome unigenes using BLASTN (e ≤ 1.00 × 10^−7^). Only 32.88% ESTs (14,512,) from GenBank matched to 23,874 unigenes (Additional file 2). This was probably associated with different tissues used for transcriptome analysis. It also highlights the high level of sequencing depth achievable through NGS compared to low coverage obtained using conventional cDNA library sequencing. Furthermore, 97,379 novel yam unigenes were discovered, some of which may be specifically expressed in yam tuber tissue. These novel unigenes may serve as a crucial genomic resource for future studies, such as gene identification, cloning and functional analysis.

### Annotation, functional classification and KEGG pathway analysis of the unigenes

To acquire the most informative and complete annotation, all assembled unigene sequences were matched against the NCBI non-redundant protein (NR), the *Arabidopsis thaliana* protein dataset of NR (ATNR), Gene Ontology (GO), and the Kyoto Encyclopedia of Genes and Genomes (KEGG) by BLASTX (e ≤ 1.00 × 10^−5^). Out of the 125,123 unigenes, 60,020 (49.5%) represented significant match to genes encoding proteins or putative function in at least one of these public databases (Table 2), whereas 50.5% unigenes could not be annotated to predicted coding regions with unknown functions in other species. In comparison with previous publications for yam and other non-model plant species [31,32], the low rate of annotated unigenes indicated that assembled unigenes, particularly sequences without a significant homologous hit, were potentially novel gene sequences not yet reported in other crops.

**Table 2:** **Summary statistics of functional annotation for yam tuber unigenes in public databases**

**Public protein database**	**NO. of unigene hit**	**Percentage (%)**
NR	58,439	48.2
ATNR	50,579	41.7
GO	43,594	35.9
KEGG	24,289	20.0
Total	60,020	49.5

**Figure 2:**
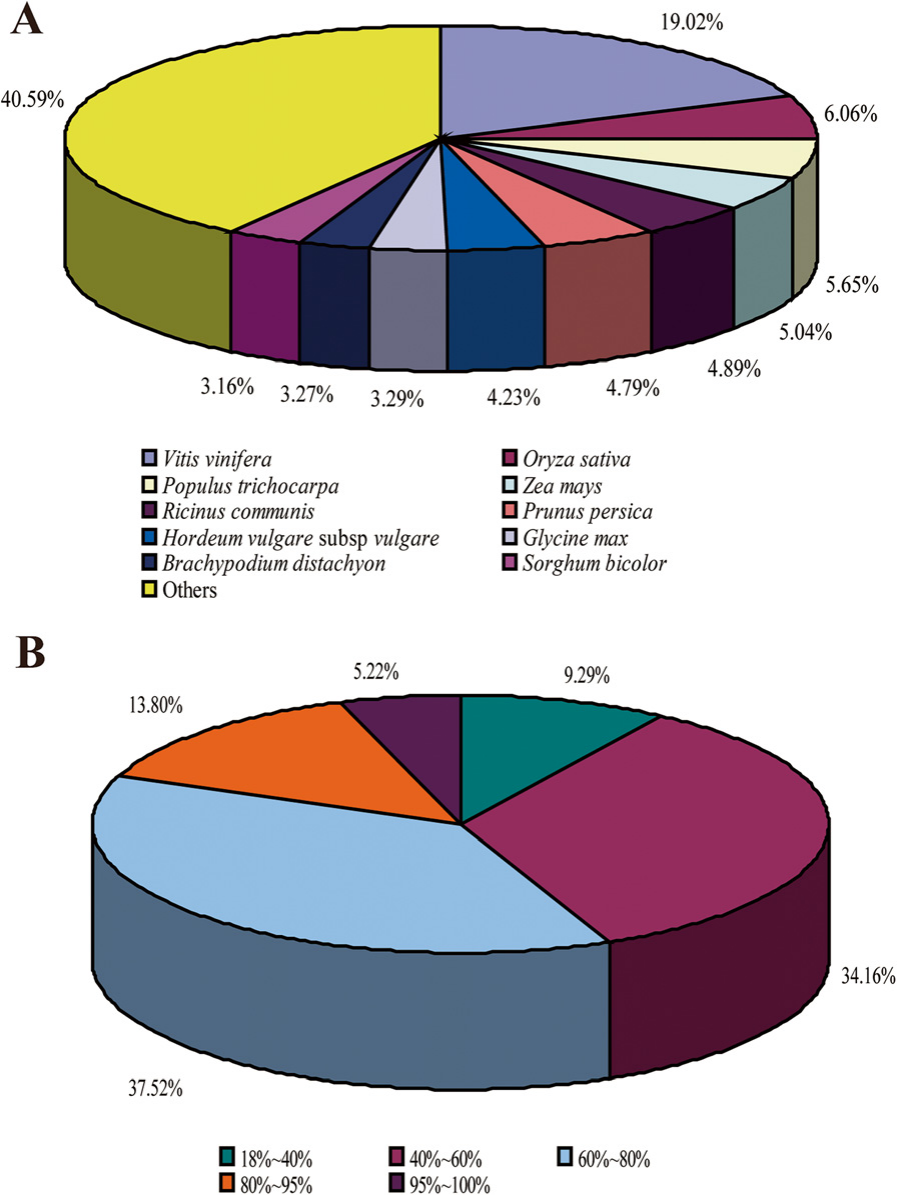
Sequence homology of yam tuber sequences BLASTED against NCBI non-redundant (NR) database. **A**: Species distribution of the top BLAST hits; **B**: Similarity distribution of top BLAST hits for each unigene.

For unigene sequences in the NR annotations, Blast search analysis further revealed that a total of 11,115, 3,542, 3,304, 2,943, 2,859 unigenes respectively matched with the sequences from *Vitis vinifera*, *Oryza sativa*, *Populus trichocarpa*, *Zea mays*, and *Ricinus communis* with the highest homology (Figure 2A). Similar distributions were also observed for yam in previous study [21]. Moreover, the identifying distribution pattern showed that 19.02% of the sequences had a similarity higher than 80%, while 71.68% showed a moderate similarity (40-80%), and the remaining 9.29% showed a lower similarity (18-40%) (Figure 2B).

**Figure 3:**
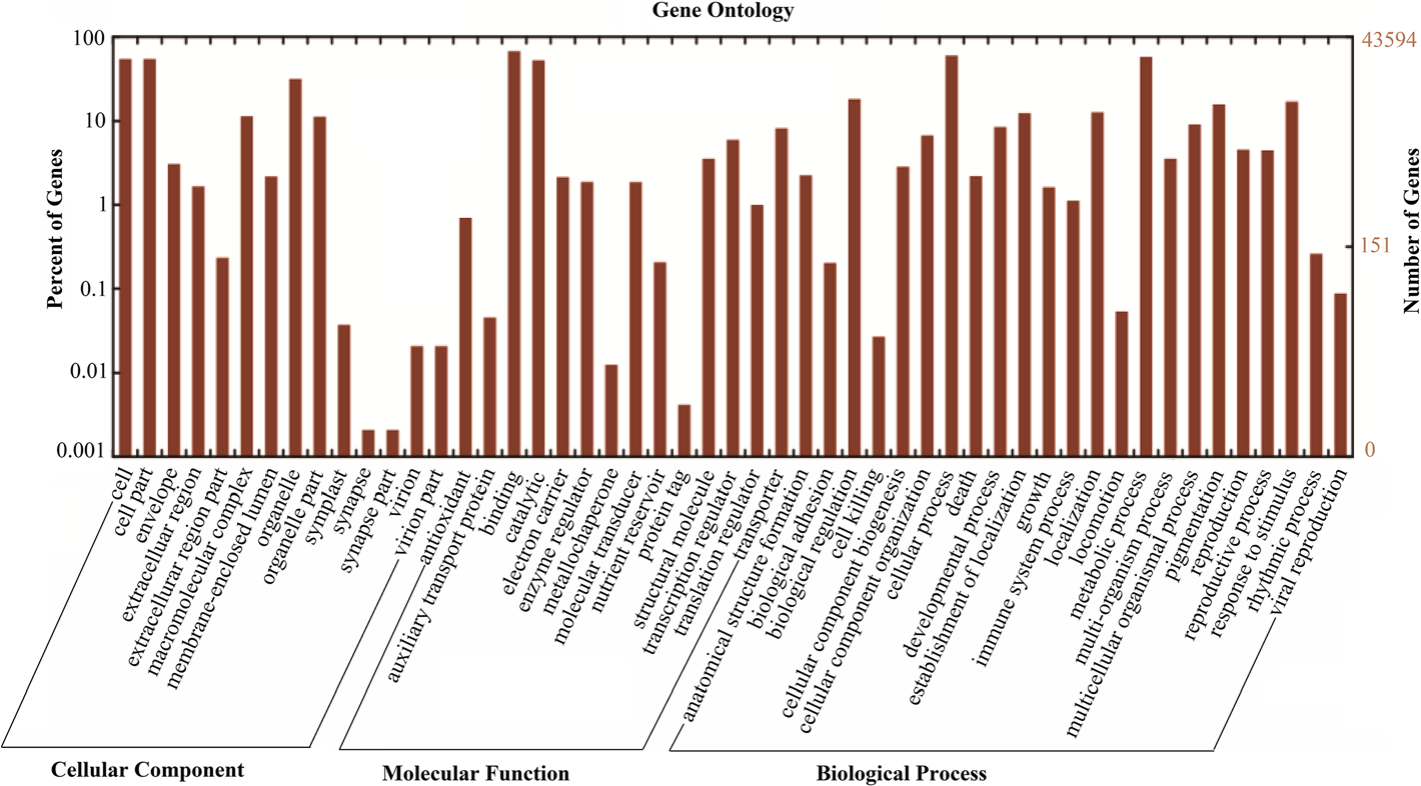
Gene ontology (GO) functional annotation of unigenes. All 43,594 unigenes are classified into 51 functional terms: 23 terms in biological process, 14 terms in cellular component, and 14 terms in molecular function.

When describing the properties of genes and their products, their functional classification is the most important. In this study, GO functional analysis was performed using Blast2GO to characterize all assembled unigenes; the result is shown in Figure 3. A total of 43,594 unigenes were classified into 51 functional terms, including 23 terms in biological process, 14 terms in cellular component, and 14 terms in molecular function. Within biological process, “cellular process” (GO:0009989) with 28,608 unigenes and “metabolic process” (GO:0008152) with 27,564 unigenes were predominant. Under the cellular component, the “cell” (GO:0005623, 25,988 unigenes), “cell part” (GO:0044464, 25,988 unigenes), and “organelle” (GO:0043226, 15,029 unigenes) represented the majority of this category. Similarly, for molecular function, the terms of “binding” (GO:0005488, 32,229 unigenes) and “catalytic activity” (GO:0003824, 25,042 unigenes) were the most abundant assigned terms. These GO annotations demonstrated that the unigenes expressed in yam tuber encode diverse structural, regulatory and stress response proteins.

**Figure 4:**
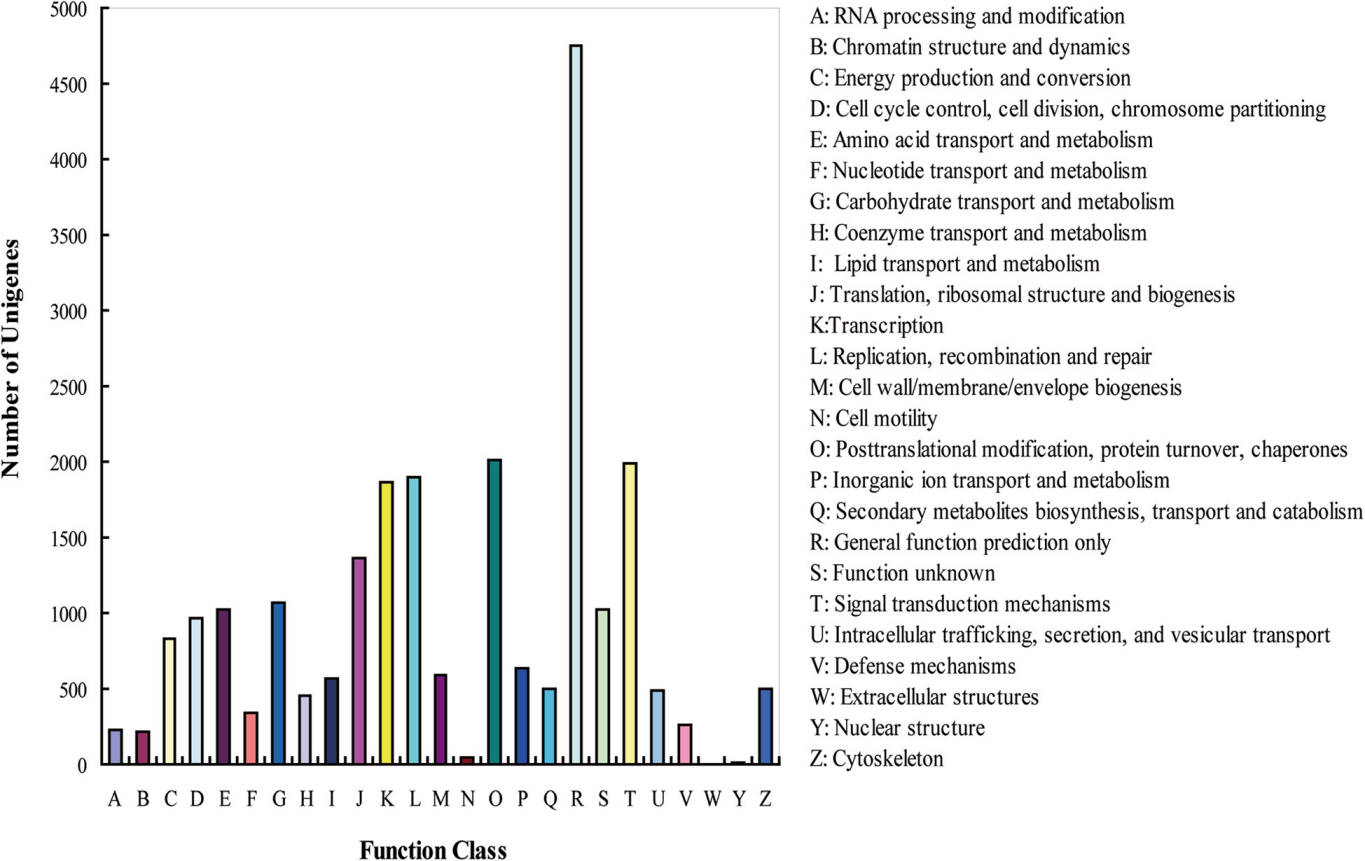
COG function classification of the yam tuber transcriptome. A total of 23,633 unigenes showing significant homology to the COGs database within NCBI (E-value ≤ 1.0 e^−5^) were classified into 25 categories.

Furthermore, all annotated unigene sequences were matched against Cluster of Orthologous Groups (COG) database to predict and classify possible functions. Out of 58,439 NR hits, a total of 23,633 sequences with COG annotations were assigned into 25 COG categories (Figure 4). The “general function prediction only” category represented the largest group (4,747), followed by “posttranslational modification”, “protein turnover and chaperones” (2,011), “signal transduction mechanisms” (1,992), “replication, recombination and repair” (1,898) and “Transcription” (1,869), and only one sequence was assigned into extracellular structures.

In addition, to identify which metabolic pathways were enriched, a pathway-based analysis was conducted through the KEGG pathway database using BLASTX with an E-value cutoff of <10^−5^. In total, 24,289 unigenes were assigned to 279 KEGG pathways (Additional file 3). The result reveals that metabolic pathway (Ko01100) was the most enriched (4,064 unigenes), followed by biosynthesis of secondary metabolites (Ko1110; 1,687 unigenes) and microbial metabolism in diverse environments (Ko1120; 1,020 unigenes). The focus of this study was differential anthocyanin accumulation in the DP cultivar. Therefore, genes associated with two secondary metabolic pathways including flavonoid biosynthesis, flavone and flavonol biosynthesis were separately analyzed. A total of 61 genes were found to be directly or indirectly involved in flavonoid biosynthesis, and they were mapped and highlighted in this pathway (Ko00941) (Additional file 4). In contrast, relatively few genes (25) were found to encode key enzymes in the flavone and flavonol biosynthesis (Ko00944) (Additional file 5). Overall, these findings provide useful information to further uncover the molecular mechanism of anthocyanin accumulation in yam tuber.

### Identification of differentially expressed genes (DEGs) in yam tubers of the purple-flesh and white-flesh cultivars

To profile gene expression, the expression levels were measured as Fragments Per Kilobase of transcript per Million fragments mapped (FPKM), with FPKM values ranging from 0 to 10^4^ [33]. As a result, 63,040 and 100,140 unigenes were discovered in the DP and DW libraries, respectively. Among them, 15,048 unigenes specifically expressed in the tuber of DP, 52,148 unigenes only expressed in the DW, and 47,992 unigenes expressed in both cultivars. This indicated that some unique genes may play an important role in the accumulation of purple pigment.

Based on the false discovery rate (FDR) ≤ 0.05, and fold change (FC) ≥ 1, 511 DEGs were identified from the two libraries, among which 288 genes were up-regulated, and 223 genes were down-regulated in DP versus DW. For a detailed comparison, see Additional file 6. There were more up-regulated genes than down-regulated ones, suggesting that many genes were positively regulated for biosynthesis of anthocyanins. Similar results were also reported in other species [19,20]. Annotation of differentially expressed unigenes revealed that 433 unigenes were grouped into 45 GO groups while the remaining 78 unigenes could not be classified (not shown). The most common categories were “intracellular part” (27 up-regulated and 17 down-regulated) and “protein binding” (24 up-regulated and 17 down-regulated), followed by “cellular macromolecule metabolic process”, and “intracellular organelle”.

### Identification of candidate genes associated with the flavonoid biosynthesis pathway

**Figure 5:**
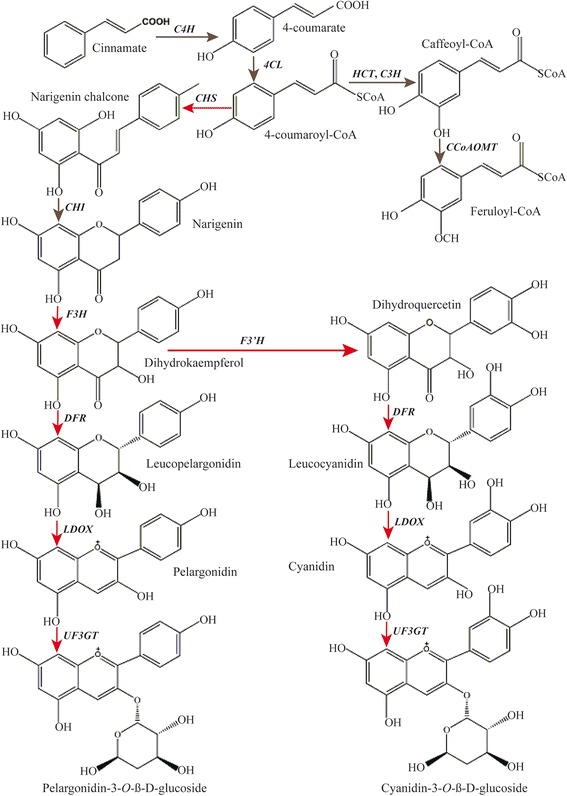
Simplified diagram depicting the flavonoid biosynthesis pathway in yam tubers. Gene abbreviations are listed in Table 3. Red arrows indicate genes that were significantly up-regulated in the purple-fleshed yam tuber. Gray indicates no change in gene expression between two tuber types.

Flavonoids are a class of important secondary metabolites including hydroxycinnamic acids, isoflavones, flavonols, phlobaphenes, pro-anthocyanidins and anthocyanins. In our annotated yam transcriptome, multiple unigenes of encoding almost all known enzymes associated with biosnythesis of anthocyanin and its derivatives in the FBP were identified (Table 3, Figure 5).

**Table 3:** **Candidate genes associated with anthocyanin pigmentation in yam tuber.**

**Gene family**	**Name**	**Target description**	**KO id (EC No.)**	**NO. all** ^**a**^	**NO. up** ^**b**^	**NO. down** ^**c**^
Phenyl-propanoid/ Flavonoid pathway	*C4H*	Trans-cinnamate 4-monooxygenase	K00487 (1.14.13.11)	5	1	0
	*C3H*	Coumaroylquinate 3′-monooxygenase	K09754 (1.14.13.36)	3	0	0
	*HCT*	Shikimate hydroxycinnamoyl transferase	K13065 (2.3.1.133)	8	0	0
	*LAR*	Leucoanthocyanidin reductase	K13081 (1.17.1.3)	1	0	0
	*ANR*	Anthocyanidin reductase	K08695 (1.3.1.77)	1	0	0
	*CHS*	Chalcone synthase	K00660 (2.3.1.74)	17	1	0
	*CHI*	Chalcone isomerase	K01859 (5.5.1.6)	1	0	0
	*F3H*	Flavanone 3-hydroxylase	K00475 (1.14.11.9)	2	1	0
	*F3’H*	Flavonoid 3′-monooxygenase	K05280 (1.14.13.21)	8	2	0
	*DFR*	Dihydroflavonol 4-reductase	K13082 (1.1.1.219)	2	1	0
	*LDOX*	Leucoanthocyanidin dioxygenase	K05277(1.14.11.19)	2	2	0
Glycosyl-transferase	*UGT75C1*	Anthocyanidin5-O-glucosyltransferase	K12338 (2.4.1.298)	1	0	0
	*UF3GT*	Flavonol 3-O-glucosyltransferase	K10757 (2.4.1.91)	3	1	0
O-methyl-transferase	*CCoAOMT*	Caffeoyl-CoAO-methyltransferase	K00588 (2.1.1.104)	11	0	0
	*FOMT*	Flavonol 3-O-methyltransferase	K05279 (2.1.1.76)	13	0	1
Glycosidase	*GUSB*	beta-Glucuronidase	K01195 (3.2.1.31)	1	0	0

^a^No. All, the total number of unigenes investigated.

^b^No. Up, the number of unigenes with expression significantly up-regulated in purple -fresh tuber of yam compared with in white one.

^c^No. Down, the number of unigenes with expression significantly down-regulated in purple-fresh tuber of yam compared with in white one.

As shown in Figure 5, flavonoids are initially derived from cinnamate and converted to chalcone via the phenylpropanoid pathway by cinnamate 4-monooxygenase (*C4H*) (EC 1.14.13.11; 5 annotated unigenes) and *CHS* (EC 2.3.1.74; 17 unigenes). Subsequently, *CHI* (EC 5.5.1.6; 1 unigene) catalyzes the stereo-specific cyclization of chalcones into naringenin. Furthermore, naringenin can be converted through *F3H* (EC 1.14.11.9; 2 unigenes) and *F3’H* (EC 1.14.13.21; 8 unigenes) to produce dihydroxyflavonols including dihydrokaempferol (DHK) and dihydroquercetin (DHQ). These flavanones serve as the lead compounds for conversion into almost all flavonoids. Following the above reaction, *DFR (*EC 1.1.1.219; 2 unigenes) further catalyzes the divergent conversion of dihydroflavonols to produce colorless procyanidins including leucopelargonidin, and leucocyanidin. They are the direct precursors for production of colored anthocyanidins (pelargonidin, and cyanidin) by *LODX* (EC1.14.11.19; 2 unigenes) catalysis. In the end, two glucosyltransferases [*UGT75C1* (EC 2.4.1.298; 1 unigene) and *UF3GT* (EC 2.4.1.91; 3 unigenes)] catalyze the glucosylation of anthocyanidins to produce stable molecules of the FBP. Notably, the formation of (−) -epiflavan 3-ols (such as epicatechin and epigallocatechin) is also achieved by a two-step conversion of leucoanthocyanidin by leucoanthocyanidin reductase [*LAR*, (EC 1.17.1.3; 1 unigene)] and anthocyanidin reductase [*ANR*, (EC 1.3.1.77; 1 unigene)] (not shown in Figure 5), suggesting that flavonoid biosynthesis looks more like a complex metabolic grid than a linear pathway [34].

Considering the anthocyanin accumulation in purple-flesh tuber is associated with specific molecular functions, we compared the differences in gene expression profile of the purple and white flesh tubers to identify putative genes co-expressed with anthocyanin accumulation. Among the above described genes involved in the FBP (Table 3), one *CHS* (unigene 003987), one *F3H* (unigene005154), one *DFR* (unigene004195), one *UF3GT* (unigene02509), two *F3’H* (unigene014794, unigene004018), and two *LDOX* (unigene028912, unigene017716) homologous sequences were significantly up-regulated in the purple-flesh tuber. In contrast, one *O*-methyltransferase (*FOMT*, unigene065894) was significantly down-regulated. These up-regulated genes code for important proteins and their expression directly correlated with anthocyanin biosynthesis (Figure 5). For example in the upstream of the FBP, the up-regulated *CHS, F3H and F3’H* in purple-flesh tubers can increase functional redundancies for forming primary precursor (chalcone) and lead compounds (DHK, DHQ) of all flavonoids. Similar results were also observed during the differential pigment deposition in potato tubers [20], peach and grape flowers [14,15].

On the other hand, in the downstream of FBP, three up-regulated genes (*DFR*, *LDOX*, *UF3GT*) also play a critical role during formation of colored anthocyanins. We found that *DFR* and *LDOX* unigenes were not expressed in the white-flesh tubers, whereas two *LDOX* unigenes were expressed at high levels in the purple-flesh tubers (Additional file 6). In addition, the up-regulated glycosyltransferase (*UF3GT*) can potentially make structural modifications to anthocyanins. Two anthocyanins (cyanidin-3- *O*-glucoside and pelargonidin-3- *O*-glucoside) in the purple-flesh tuber are glycosylated at the 3-postion of the C-ring by this enzyme. Similar results were also reported in previous studies [35-37]. For instance, two glycosyltransferases, *UGT79B1* and *UGT84A2* were found to cause high levels of anthocyanin modifications (3-O-glucosylated anthocyanidins) in *Arabidopsis* flavonoid biosynthesis [35], whilst anthocyanins were drastically reduced in the *UGT79B1* and *UGT84A2* knockout mutants. Besides, the *O*-methyltransferase is one of the most important modification reactions of flavonoids and the resulting *O*-methylated flavonoids have been shown to display new biological activities [38]. In this study, the down-regulated *O*-methyltransferase (*FOMT*) was assigned to code quercetin-3-*O*-methyltransferase protein and may have redundant function in the FBP. Taken together, these results indicate that key genes responsible for the FBP have a higher expression level in the purple-flesh tubers of yam*.* This finding is an important explanation of well-known higher antioxidant activity in pigmented tissues found in a number of tissues.

### Identification of genes associated with transcription factors (TFs)

Besides structural genes, it is well known that transcription factors play an essential role in regulating the overall activity of flavonoid biosynthesis. In most species, the anthocyanin branch within the FBP is controlled by a ternary complex of MYB-bHLH-WD40 TFs [5,39], which generally regulate expression of many structural genes. In our transcriptome database, a total of 183, 146, 95 unigenes were respectively predicted to code bHLH, MYB, and WD40 proteins including a large number of its members. Of these genes, the transcriptomic analysis detected four TFs that were differentially expressed between the two cultivars of yam tubers, including three WD40 repeat proteins with one up-regulated (unigene050252) and two down-regulated (unigene041043, unigene056944) in purple-flesh tubers. Further, one MYB4R1 protein (unigene029894) was also found to be up-regulated in purple-flesh tubers (Additional file 6). The high variation in expression of structural genes associated with the FBP in the purple-flesh tubers may most likely be regulated by one or more of these TFs. However, the specific function of these TFs in the FBP of yams still needs to be validated using a functional genomics approach.

### Gene validation and expression analysis

**Figure 6:**
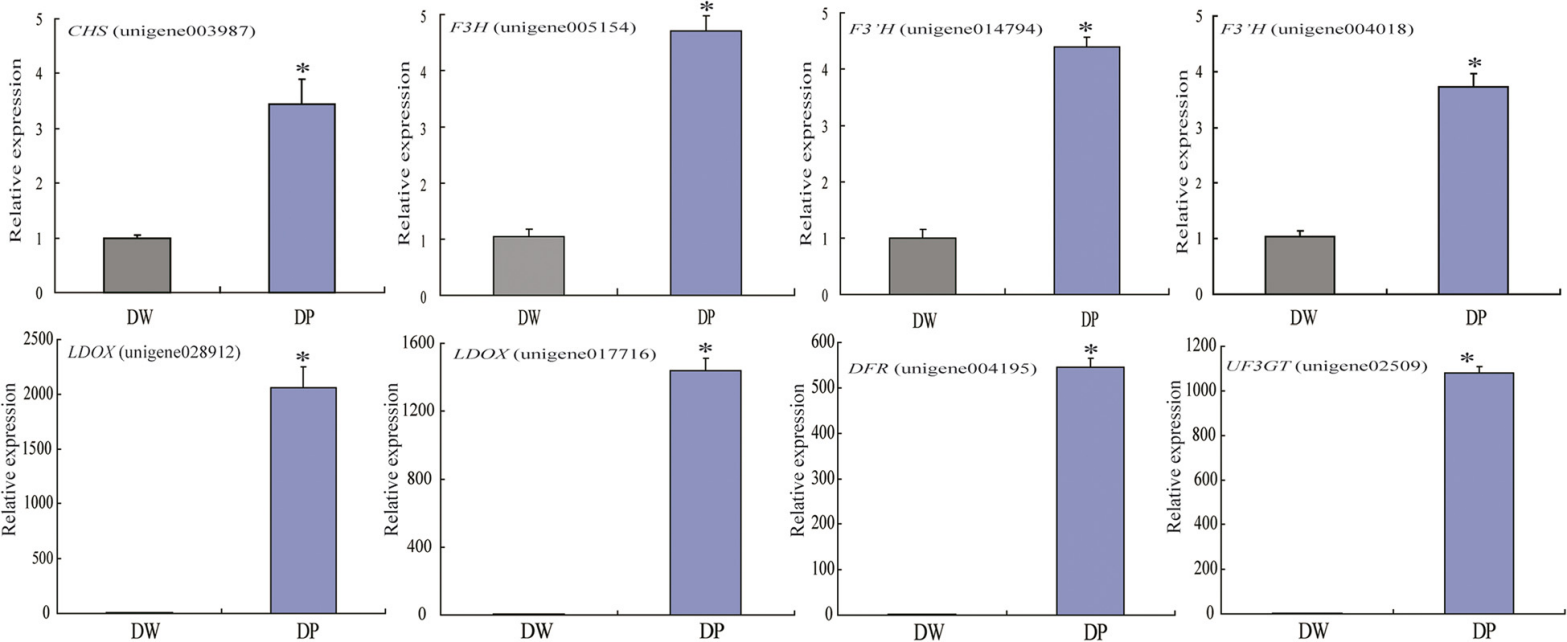
qRT-PCR analysis of eight differentially expressed unigenes associated with the FBP. Error bars were calculated based on three replicates. UBC was used as an internal control for normalization. DW = white-flesh tuber; DP = purple-flesh tuber.

It was reported that several genes involved in the FBP showed special expression patterns in different species such as *CHS*, *F3H*, *DFR*, *LDOX* genes in *Brassica juncea* Seed Coat [19], *Solanum tuberosum* L. tuber [20], *Carthamus tinctorius* L flower [40], *Magnolia sprengeri* pamp flower [41]. Therefore, to experimentally confirm that the unigenes obtained in this study from transcriptome analysis were indeed differentially expressed, eight DEGs (Additional file 7) associated with the FBP were chosen for real-time quantitative PCR assay. The expression profiles of these unigenes are shown in Figure 6. Results showed that unigene 003987 (*CHS*), unigene005154 (*F3H*), unigene014794 and unigene004018 (*F3’H*), unigene004195 (*DFR*), unigene028912 and unigene017716 (*LDOX*), and unigene02509 (*UF3GT*) were up-regulated in the purple-fleshed tuber of yam, which was well consistent with those observed by transcriptome analylsis (Table 3, Figure 5). This result further confirms the reliability of RNA-seq analysis.

### Identification of simple sequence repeats (SSRs) in yam

Usually, gene-derived SSRs are more transferable between species than random genomic SSRs. This is perhaps because they are associated with functional genetic variation, as opposed to non-coding SSRs, with presence in transcribed regions potentially influencing gene function, transcription or translation [26]. In this study, transcriptome analysis of the two yam tuber cultivars (DP and DW) led to identification of 11,793 SSRs within 121,253 unigenes, of which, 977 sequences contained more than 1 SSR, and 1706 SSRs were present in compound form. The observed frequency of unigenes was 8.6% (10,426); considering that approximately 71,983 kb total size was analyzed, and least one SSR per 6.1 kb could be detected in the expressed sequences of yam.

The motifs of 11,793 SSRs contained 5,788 (49.08%) dinucleotides, 5,582 (47.33%) trinucleotides, 335 (2.85%) tetranucleotides, 44 (0.37%) pentanucleotides and 44 (0.37%) hexaucleotides (Table 4). The most abundant repeat type was AG/CT, followed by AAG/CTT, and AAAT/ATTT. Further, 6,082 SSR primer pairs were successfully designed using Primer 3. The details of the frequency of SSR motif and genic-SSR primers sequences (including designing parameters) are summarized in Additional file 8. Very recently, we revealed that Chinese yam species have rich genetic diversity and phenotype traits including stable tuber yield, taste, texture, and dry matter content [28]. In comparison with previous study using Roche 454 sequencing technology [21], a larger number of new genic-SSR markers were developed in this study, and they may be closely linked to these qualitative traits. In the future, these functional gene-based markers, will make it possible to construct a high density linkage map or association map for identification of quantitative traits loci (QTL) associated with tuber quality traits in yams.

**Table 4:** **Frequencies of repeat types with repeat number in the cSSRs of yam**

**Motif length**	**Repeat number**							**Total**	**%**
	**5**	**6**	**7**	**8**	**9**	**10**	**>10**		
Dinucleotides	-	1,530	1,052	813	1,029	933	431	5,788	49.08
Trinucleotides	2,729	1,608	1,020	123	48	20	34	5,582	47.33
Tetranucleotides	272	48	12	1	1	0	1	335	2.85
Pentanucleotides	43	1	0	0	0	0	0	44	0.37
Hexaucleotides	22	14	3	5	0	0	0	44	0.37
Total	3,066	3,201	2,087	942	1,078	953	466	11,793	-
%	26.00	27.14	17.69	7.99	9.14	8.08	3.95	-	-

## Conclusions

The focus of this study was use of NGS-based Illumina paired-end sequencing platform to characterize the gene expression differences between an elite purple-flesh tuber and conventional white-flesh tuber of yam. A total of 125,123 unigenes were identified from the two cDNA libraries, which will contribute significantly to further genome-wide research and analyses of this species and other related species. Analysis of the transcriptome data revealed a number of candidate genes which are possibly involved in purple-flesh tuber formation. The candidates include not only structural genes such as *CHS*, *F3H*, *F3’H*, *DFR*, *LDOX* and *UF3GT*, but also some transcription factors (bHLH, MYB, and WD40) that potentially regulate development of purple-flesh in yam tubers. Such knowledge can be used to genetically enhance tuber color of conventional white-flesh cultivar. In addition, we also used transcriptomic data as a resource to develop new SSR markers. These marker sets will facilitate identification of quantitative traits loci (QTL) associated with yam tuber quality in future.

## Methods

### Plant materials

The elite purple-flesh tubers and the conventional white-flesh tubers of yam (*D. alata*) were cultivated in a yam producing region (Wenzhou city, Zhejiang province, China; 121°09′48.82″ E and 28°27′53.62 N). Both were planted at the same time and cultivated in similar conditions. Tubers were harvested 10 days after new tuber emergence (DAM) and used for transcriptome analysis. Tubers of each cultivar were collected from five different plants, with a total of 15 tubers per cultivar. The tubers were washed and their skin was peeled off. The samples were labeled as DP (purple-flesh tuber) and DW (white-flesh tuber), then immediately frozen in liquid nitrogen, and stored at −80°C prior to use.

### RNA extraction, cDNA library construction and Illumina deep sequencing

Total RNA from the DP and DW samples was extracted using the RNAiso kit for polysaccharide-rich plant tissue (Takara Biotechnology (Dalian) Co., Ltd.) and purified using RNeasy plant mini kit (Qiagen, Valencia, CA) to avoid DNA contamination. The RNA quality was analysed by measuring the absorbance at 260 nm/280 nm (A260/A280) using a ND-1000 spectrophotometer (Nano-Drop Technologies, Wilmington, DE, USA). Further, RNA Integrity Number (RIN) values were determined using a Bioanalyzer 2100 (Aligent Technologies, Santa Clara, CA) to make sure all samples had a RIN greater than 8.5. Two separate RNA pools for the DP and DW cultivars were prepared for cDNA library construction, each comprising 15 RNA samples from 15 tubers of five plants per cultivar.

Two sequencing libraries were constructed using a cDNA Synthesis kit (Illumina Inc., San Digo, CA, USA) following the manufacturer’s instructions. Paired-end (2 × 150 bp) sequencing of the cDNA libraries was performed on the Illumina HiSeq 2000 (Illumina Inc., San Diego, CA, USA). Libraries from both the cultivars yielded more than 4 GB of clean data. Sequencing was completed by the Hangzhou Woosen Bio-technology Co. Ltd. (Hangzhou, China).

### Reads assembly and transcriptome annotation

The clean reads were obtained by read trimming of raw data by removing adaptors, reads in which unknown bases were more than 10%, low-quality reads with quality scores less than Q30, and low-quality bases less than (Q30) at the 3′ end. Next, the high-quality filtered reads were further assembled using a *de novo* assembly program Trinity (released 2011-05-19, http://trinityrnaseq.sourceforge.net/) with the main parameters “K-mer = 25, group_pairs_distance = 500, min_glue = 2, min_kmer_cov = 1” [42]. ^.^Briefly, for each library (DP and DW), short reads were first assembled into longer contiguous sequences (contigs) according to their overlap regions, and then these reads were mapped back to the contigs based on their paired-end information. With paired-end reads it is possible to detect the contigs from the same transcript as well as the distances between these contigs. Afterwards, the contigs were further assembled, and the assembled sequences that could not be extended on either end and were defined as unigenes. Finally, the potential unigenes from DP and DW library were clustered using the TGICL clustering tool [43] to acquire a single set of non-redundant unigenes. In addition, to obtain assembly statistics profile about reads that could be mapped back to the assembled unigenes, TopHat (version 2.0.8) (released 2013-02-26, http://tophat.cbcb.umd.edu/) [44] with the parameter “mate-inner-dist = 250”, was used to align short reads to the constructed transcripts by *de novo* assembling.

All assembled unigenes were annotated by matching against the NCBI non-redundant protein (NR), the *Arabidopsis thaliana* protein dataset of NR (ATNR), Gene Ontology (GO), and the Kyoto Encyclopedia of Genes and Genomes (KEGG) using the BLASTX analysis with a cut-off E-value of 10^−5^. Based on NR annotation, the Blast2GO software (version 2.3.5) was used to obtain GO annotations according to molecular function, biological process and cellular component ontologies (http://www.geneontology.org) [45]. The unigene sequences were subsequently matched against the COG database to predict and classify possible functions. The KEGG pathway annotation was also performed by comparison against the KEGG database using the online KEGG Automatic Server (KAAS) (http://www.genome.jp/tools/kaas/) [46,47].

### Differentially expressed genes (DEGs) between the DP and DW tubers

In order to assess the differential expression between the two investigated yam cultivars, TopHat (version 2.0.8) was first used to match against the assembled unigenes, which was followed by estimation of total mapped reads [44]. After the alignment, cufflinks (version 2.1.1) (released 2013-04-11, http://cufflinks.cbcb.umd.edu) was used to estimate the abundances of unigenes as Fragments Per Kilobase of transcript per Million fragments mapped (FPKM) [33]; and cuffdiff was carried out to perform pairwise comparisons between different investigated cultivars. Differentially expressed genes (DEGs) were further characterized and estimated using the R software module edge R (R v2.14; edgeRv 2.3.52) in term of the results from cufflinks [48]. False discovery rate (FDR) <0.05 and an estimated absolute log_2_ fold-change (log_2_ FC) ≥ 1 were used as threshold for determining significant difference in gene expression between the purple- and white flesh tubers of yam. Moreover, all DEGs were mapped to terms in the KEGG database and searched for KEGG terms to identify pathways related to purple-flesh trait in yam tubers.

### qRT-PCR verification

Total RNA was extracted from the white and purple flesh-tubers of yam as described above. Approximately 2 mg of total RNA per sample was treated with DNaseI (Takara), and reverse transcribed into cDNA using Promega A3500 reverse transcription system. Eight DEGs were selected for Quantitative real-time PCR (qRT-PCR) analysis to verify the expression patterns revealed by the RNA-seq analysis. Gene specific qRT-PCR primers (Additional file 7) were designed using Premier 5.0 software (Premier Biosoft International, Palo Alto, CA). qRT-PCR was performed using SybrGreen qRT-PCR Master Mix (Ruian Biotechnologies, Shanghai, China) in an ABI 7500 FAST Real-Time PCR System (Applied Biosystems, Foster City, CA, USA). Amplification program comprised an initial denaturation step at 95°C for 2 min, followed by 40 cycles of denaturation at 95°C for 10 s and annealing at 60°C for 30 s. Three replicates were performed, and the amplicons were subject to melting curve analysis to determine amplification specificity. The relative expression level of the selected unigenes were normalized to UBC gene and calculated using the 2^-ΔΔCt^ method [49].

### Simple sequence repeats (SSRs) identification and primer design

Simple sequence repeats (SSRs) in unigene sequences were identified using MIcroSAtellite package (MISA, http://pgrc.ipk-gatersleben.de/misa). The SSR search parameters were defined to identify di-, tri-, tetra-, penta- and hexa-nucleotide motifs with a minimum of 6, 5, 4, 5 and 5 repeats, respectively. Subsequently, primer pairs were designed for genes with SSRs using the Primer3 (version 2.23) (http://sourceforge.net/projects/primer3/) with default settings [50], and the PCR product size ranging from 100 to 280 bp.

### Availability of supporting data

All clean reads generated by Illumina sequencing have been deposited in the Sequence Read Archive (SRA) database (http://trace.ncbi.nlm.nih.gov/Traces/sra/) under the accession ID SRX652481 for DP, and SRX652483 for DW.

## Additional files

Additional file 1:
**Length distribution of unigenes within the yam tuber transcriptome.**
Additional file 2:
**Comparison of yam unigenes from this study with ESTs obtained from Genbank.**
Additional file 3:
**KEGG pathways for the assembled yam tuber unigenes.**
Additional file 4:
**Schematic representation of the flavonoid biosynthesis pathway.** Each box represents a gene encoding a key enzyme involved in flavonoid biosynthesis. Numbers in each box are EC codes of each gene. Genes in red boxes represent those captured by our unigenes, and their expression values (FPKM) are higher than 10. Other colored and uncolored boxes indicate undetected genes. EC code definitions can be found at: http://www.genome.jp/kegg-bin/show_pathway?map00941. Additional file 5:
**Schematic representation of the flavone and flavonol biosynthesis pathway.** Each box represents a structural gene encoding a key enzyme involved in flavones and flavonol biosynthesis. Numbers in each box are EC codes of each gene. Genes in red and green boxes represent those captured by our sequence, with red boxes exhibiting genes expressed higher than 10, and green box genes with expression values (FPKM) less than 10. Other colored and uncolored boxes exhibit undetected genes. EC code definitions can be found at: http://www.genome.jp/kegg-bin/show_pathway?map00944.
Additional file 6:
**Genes differentially expressed between the purple-flesh and white-flesh tubers of yam.** Structural genes and transcription factors related to flavonoid biosynthesis are highlighted in red. Additional file 7:
**Primers used for qRT-PCR analysis.** Primers were designed using the Primer Premier program (version 5.0). Additional file 8:
**Simple Sequence Repeats (SSR) generated in this project.** The frequency of SSR motifs in yam unigenes, and detailed information on primers for the 6082 SSRs are respectively presented on sheet 1, and 2 of this file.
