# Supplementary figures and images for: Transciptome analysis reveals flavonoid biosynthesis regulation and simple sequence repeats in yam (Dioscorea alata L.) tubers
Source: BMC Genomics. 2015 Apr 30;16(1):346. doi: 10.1186/s12864-015-1547-8 (PMC4415240; doi:10.1186/s12864-015-1547-8)

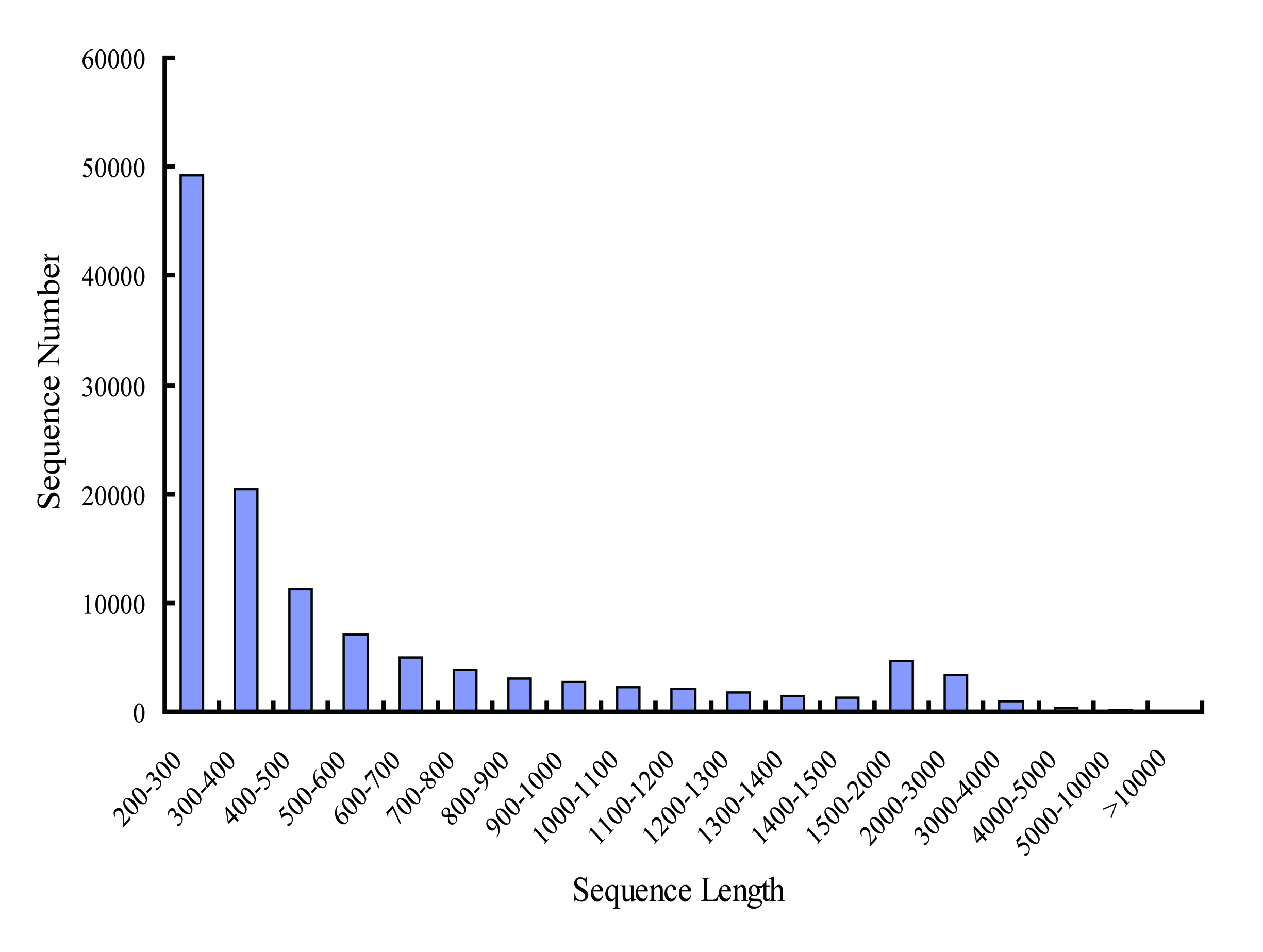

Supplement: Additional file 1: — Length distribution of unigenes within the yam tuber transcriptome. [file 12864_2015_1547_MOESM1_ESM.tiff]

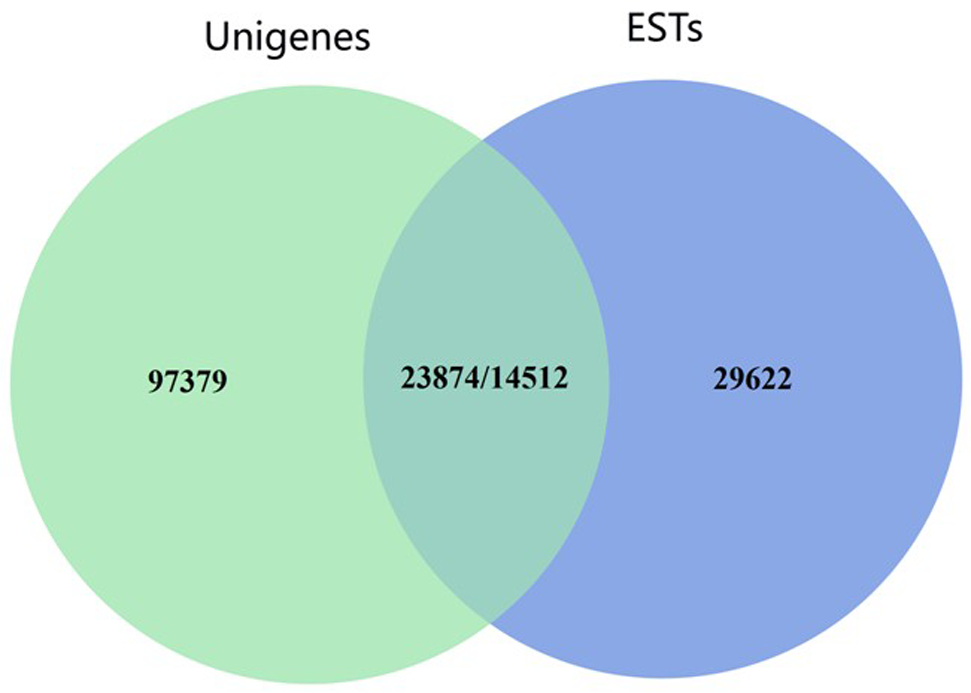

Supplement: Additional file 2: — Comparison of yam unigenes from this study with ESTs obtained from Genbank. [file 12864_2015_1547_MOESM2_ESM.tiff]

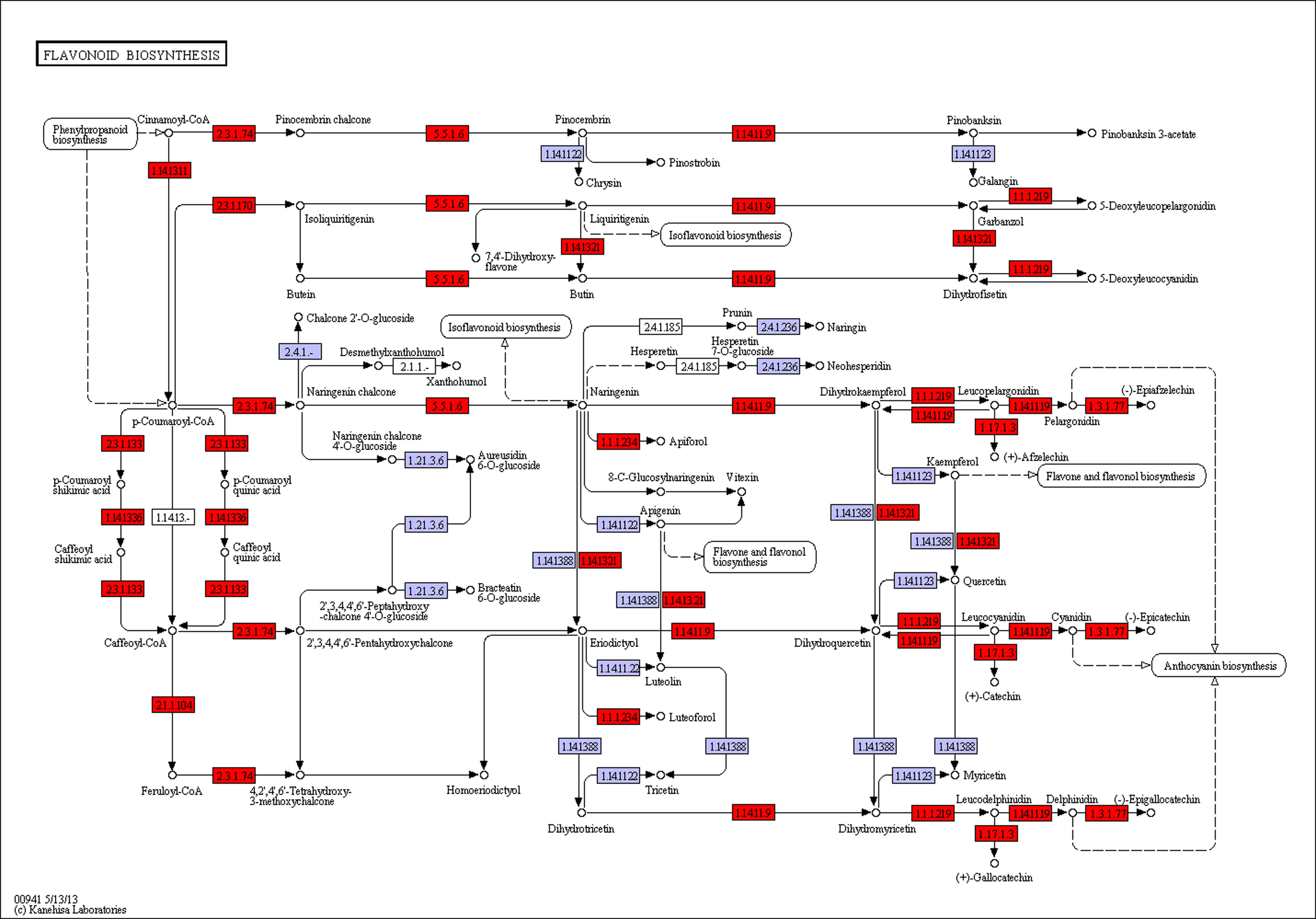

Supplement: Additional file 4: — Schematic representation of the flavonoid biosynthesis pathway. Each box represents a gene encoding a key enzyme involved in flavonoid biosynthesis. Numbers in each box are EC codes of each gene. Genes in red boxes represent those captured by our unigenes, and their expression values (FPKM) are higher than 10. Other colored and uncolored boxes indicate undetected genes. EC code definitions can be found at: http://www.genome.jp/kegg-bin/show_pathway?map00941. [file 12864_2015_1547_MOESM4_ESM.tiff]

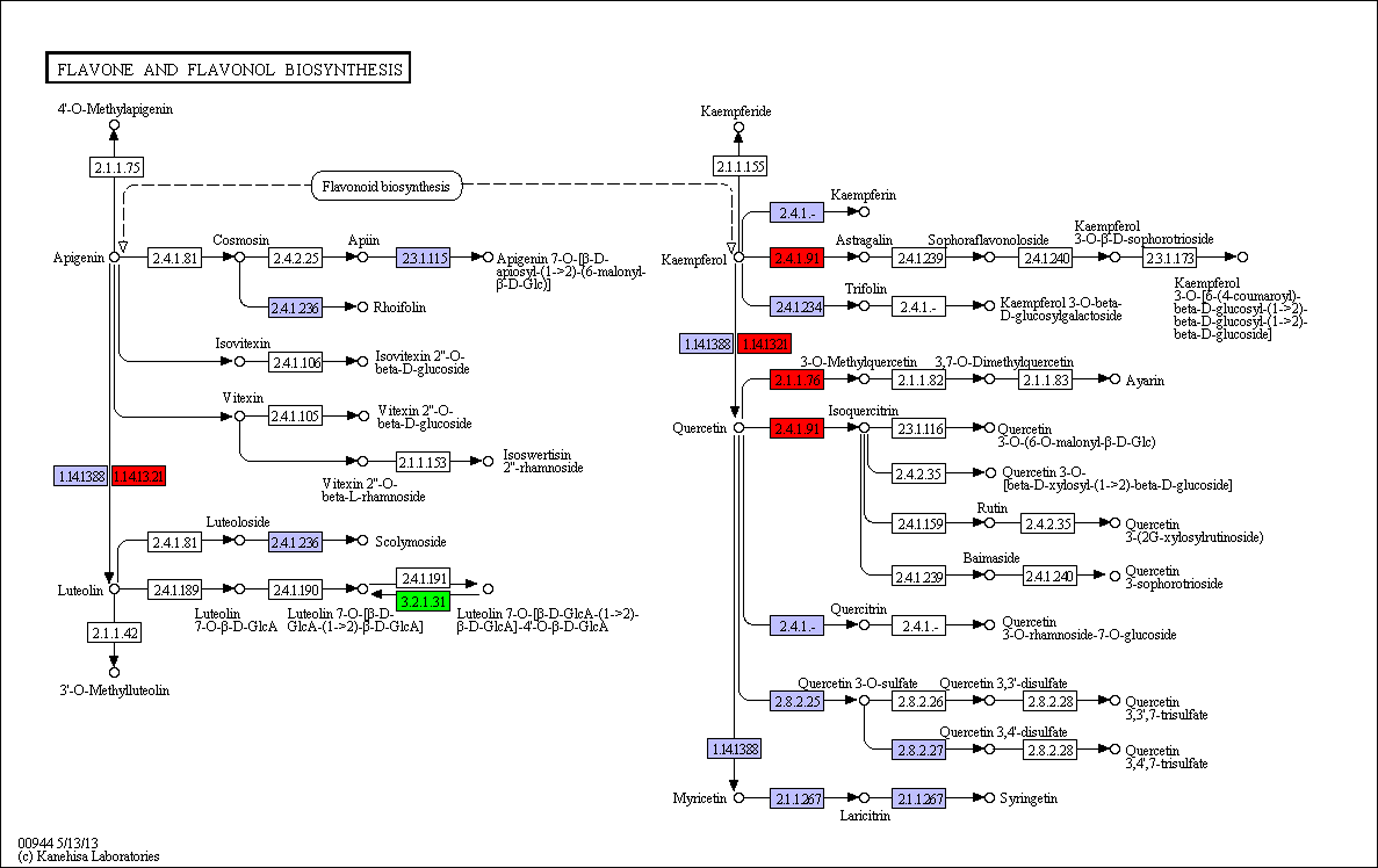

Supplement: Additional file 5: — Schematic representation of the flavone and flavonol biosynthesis pathway. Each box represents a structural gene encoding a key enzyme involved in flavones and flavonol biosynthesis. Numbers in each box are EC codes of each gene. Genes in red and green boxes represent those captured by our sequence, with red boxes exhibiting genes expressed higher than 10, and green box genes with expression values (FPKM) less than 10. Other colored and uncolored boxes exhibit undetected genes. EC code definitions can be found at: http://www.genome.jp/kegg-bin/show_pathway?map00944. [file 12864_2015_1547_MOESM5_ESM.tiff]
