# Supplementary material for: Transciptome analysis reveals flavonoid biosynthesis regulation and simple sequence repeats in yam (Dioscorea alata L.) tubers
Source: BMC Genomics. 2015 Apr 30;16(1):346. doi: 10.1186/s12864-015-1547-8 (PMC4415240; doi:10.1186/s12864-015-1547-8)
Supplement: Additional file 7: — Primers used for qRT-PCR analysis. Primers were designed using the Primer Premier program (version 5.0). [file 12864_2015_1547_MOESM7_ESM.doc]

**Additional file 7** Gene-sequences for detection by Quantitative real-time PCR

| **Gene** | **Accession No.** | **Forward primer (5’-3’)** | **Reverse primer (5’-3’)** |
| --- | --- | --- | --- |
| *CHS* | unigene003987 | TTTACCAAGCCGAGTATCCA | TCCGAGCAGACAACAAGAAC |
| *F3H* | unigene005154 | GGGACTGGAGGGAAATAG | TCAAGCCAAGAGTGAGGT |
| *F3’H* | unigene014794 | TCAACCAAGGCACAAAGT | TCCAACAGACCTCCAAAC |
|  | unigene004018 | CACCTGGTCTGACATCCG | TTAGCCTGAATCCCTTGC |
| *DFR* | unigene004195 | ATGGAAAGCAGACTTGGGAGAA | AGGGTGGAATCACTGTAATGAGGT |
| *LDOX* | unigene028912 | TTGTGGAGGATGAAGGATAGGT | GATACAAGGGTATGGGAGCAAA |
|  | unigene017716 | CCCCAGATACCGACCATT | CACTCAAGACGCCCAGAA |
| *UF3GT* | unigene025092 | GCTAAGTGTCCCAATACCTG | ACCTTTAAGCCATTTGTTTC |
| *UBC* |  | ACCCTCCACTTGGTCCTC | TGAATCTTGGCCTTCACG |
